# Molecular stratification of the human fetal vaginal epithelium by spatial transcriptome analysis: Spatial transcriptome analysis of the human fetal vaginal epithelium

**DOI:** 10.3724/abbs.2024063

**Published:** 2024-04-25

**Authors:** Ziying Ye, Peipei Jiang, Qi Zhu, Zhongrui Pei, Yali Hu, Guangfeng Zhao

**Affiliations:** 1 Department of Obstetrics and Gynecology Affiliated Drum Tower Hospital Medical School Nanjing University Nanjing 210008 China; 2 Nanjing Drum Tower Hospital Clinical College of Traditional Chinese and Western Medicine Nanjing University of Chinese Medicine Nanjing 210008 China

**Keywords:** spatial transcriptomics, vaginal epithelium, keratin

## Abstract

The human vaginal epithelium is a crucial component of numerous reproductive processes and serves as a vital protective barrier against pathogenic invasion. Despite its significance, a comprehensive exploration of its molecular profiles, including molecular expression and distribution across its multiple layers, has not been performed. In this study, we perform a spatial transcriptomic analysis within the vaginal wall of human fetuses to fill this knowledge gap. We successfully categorize the vaginal epithelium into four distinct zones based on transcriptomic profiles and anatomical features. This approach reveals unique transcriptomic signatures within these regions, allowing us to identify differentially expressed genes and uncover novel markers for distinct regions of the vaginal epithelium. Additionally, our findings highlight the varied expressions of keratin ( *KRT*) genes across different zones of the vaginal epithelium, with a gradual shift in expression patterns observed from the basal layer to the surface/superficial layer. This suggests a potential differentiation trajectory of the human vaginal epithelium, shedding light on the dynamic nature of this tissue. Furthermore, abundant biological processes are found to be enriched in the basal zone by KEGG pathway analysis, indicating an active state of the basal zone cells. Subsequently, the expressions of latent stem cell markers in the basal zone are identified. In summary, our research provides a crucial understanding of human vaginal epithelial cells and the complex mechanisms of the vaginal mucosa, with potential applications in vaginal reconstruction and drug delivery, making this atlas a valuable tool for future research in women’s health and reproductive medicine.

## Introduction

In women of reproductive age, the vaginal epithelium is a unique, estrogen-sensitive, non-keratinized stratified squamous epithelium that rests atop the lamina propria. This epithelium is composed of four distinct layers: a cuboidal basal layer, a mitotically active parabasal layer, a glycogen-rich intermediate layer, and a flattened non-cornified superficial layer characterized by pyknotic nuclei [1]. This complex structure serves a pivotal function within the female reproductive system, acting as a crucial barrier against pathogen invasions and playing essential roles in reproductive processes such as childbirth, menstruation, and intercourse [2]. Remarkably, the vaginal epithelium has also been reported to exhibit high permeability [ 1, 3] , a characteristic that has been harnessed for the delivery of medications. This unique property makes the vaginal epithelium a promising target for drug delivery systems, as demonstrated in recent studies [ 4, 5] .

Despite its pivotal role in female reproductive health, our understanding of the human vaginal epithelium remains incomplete. Previous investigations into its characteristics primarily relied on histological and immunohistochemical (IHC) techniques [ 1, 6– 8] , which identified key markers but did not provide sufficient dynamic spatiotemporal information on the epithelial differentiation process. In recent years, genetic rodent models leveraging advanced gene editing techniques, alongside surgically induced ovariectomy (OVX) models in mice and rats, have shed new light on the differentiation mechanisms of the female reproductive tract epithelium [ 9– 11] . These studies have been complemented by technological advancements such as single-cell RNA sequencing (RNA-seq) and lineage tracing, which revealed that NGFR ^+^Axin2 ^+^ cells residing in the basal layer of the mouse vaginal epithelium undergo expansion to eventually form the entire epithelium [12]. The maintenance of vaginal epithelial homeostasis is a delicate balance achieved through continuous stem cell development, culminating in shedding or degradation of cells by the immune system [ 13, 14] . While these rodent models provide valuable insights, it is essential to acknowledge the inherent differences between rodent and human vaginal epithelia [15]. Therefore, a comprehensive and systematic study of the human vaginal epithelium is imperative to elucidate whether a similar developmental pattern exists, specifically regarding the continuous development of vaginal epithelial cells from the basal to the superficial layer.

With the advent of sophisticated sequencing techniques, researchers have delved into the intricacies of the vaginal wall, utilizing transcriptome sequencing to compare patients suffering from vaginal atrophy with healthy women. Their investigations have shed light on the influence of estradiol (E2) on the vaginal epithelium and its associated gene regulatory networks [ 16, 17] . Nevertheless, a caveat of this bulk RNA sequencing approach is its inability to provide a nuanced portrayal of epithelial cells. Previous efforts employing single-cell transcriptome sequencing of the vaginal wall in women with pelvic organ prolapse (POP) have focused primarily on revealing pathological mechanisms, leaving much to be desired regarding epithelial characterization [ 18, 19] . In essence, while prior research has offered incremental insights into this multifaceted organ, a comprehensive understanding of vaginal epithelial cells across various locations and differentiation stages remains elusive.

Keratin (KRT), a prominent cytoskeletal protein found in epithelial cells, not only serves as a structural component but also plays a significant role in cell proliferation, differentiation, and the maintenance of vaginal epithelial polarity and homeostasis [20]. Furthermore, as a type of epithelium, the vaginal epithelium has been reported to abundantly express keratins. The expression of keratins is known to be differentiation-dependent, tissue-specific, and often occurs in pairs [21]. Specifically, keratins 6, 7, 8, 10, 14, and 19 reportedly exhibit distinct temporal and spatial dynamics during the development of the human female reproductive tract [6]. The presence of specific keratins can serve as a marker indicating the differentiation status and maturity levels of various epithelial cells [20].

The advent of spatial transcriptomics has ushered in a new era of expression profiling, enabling the interrogation of highly specific regions within tissue sections, even at subcellular levels. This technology facilitates the correlation of expression data with morphological features, tissue type, and spatial relationships to other structures, thereby offering a more nuanced understanding of multiple tissues and organs [22]. Since there are no spatial transcriptomic data for the vagina and to construct an unbiased spatial transcriptomic atlas of the human vaginal epithelium, our study focused on collecting tissue samples from two human fetuses aborted at 22 ^+5^ and 22 ^+6^ weeks of gestation. This choice was informed by evidence suggesting that the vaginal stratified squamous epithelia of 20-week-old fetuses have attained maturity morphologically [ 7, 23] . However, it is unknown whether the fetal vaginal epithelium is mature at the molecular level. Both samples were carefully selected as cross-sections of the fetal vagina proximal to the vaginal orifice. Our findings unveil the intricate molecular traits and functionalities of vaginal epithelial stratification, emphasizing the profound cellular diversity that exists within this tissue. This groundbreaking discovery paves the way for potential advancements in vaginal reconstruction [ 24– 29] and the optimization of vaginal drug delivery systems [ 4, 5, 13] . As research in this area continues to evolve, the implications of these findings are poised to transform our understanding and treatment of vaginal health conditions.

## Materials and Methods

### Tissue processing

Vaginal tissue samples were obtained from human fetal specimens in the second trimester following an ethically approved process of selective termination of pregnancy by the Ethics Committee of Nanjing Drum Tower Hospital (ethics number: 2021-131-02). The estimated fetal age was determined based on the mother’s last menstrual period. Six fetal specimens, derived from human fetuses at 22‒24 weeks, were meticulously collected, with two specimens designated for spatial transcriptomics sequencing, one specimen for determining the time of permeation and three specimens allocated for staining validation.

The procurement of fetal specimens adhered strictly to ethical standards and was carried out with utmost care and precision. This included careful separation of the skin of the lower abdomen, meticulous disconnection of ligaments and attachments of the fetal genitourinary system from the abdominal cavity, and subsequent access to the fetal pelvic-abdominal cavity. The fetal vaginal wall was carefully dissected from surrounding structures, including the bladder, urethra, and anorectum, to ensure the integrity and quality of the tissue samples obtained ( Supplementary Figure S1A).

### Spatial transcriptomics

The tissues harvested from the fetal vaginal region underwent a preservation process that involved freezing in isopentane, followed by embedding in OCT and cryo-sectioning, tailored specifically for use with Visium Spatial slides from 10×Genomics. Afterward, H&E staining was conducted, and brightfield microscope images of the stained tissues were captured, laying the foundation for further investigation. In preparation for library construction, an optimal permeabilization time of 24 min was established using the Visium Spatial Tissue Optimization Slide kit ( Supplementary Figure S1B). During this carefully timed permeabilization, RNA molecules were captured by barcoded spots on the Visium slides. Following capture, a high-quality library was constructed, and its integrity was verified using the DNA 1000 assay kit from Agilent Technologies (Santa Clara, USA). Quantification, performed via the ABI StepOnePlus Real-Time PCR System (Life Technologies, Carlsbad, USA), preceded the initiation of the sequencing process. The cDNA library was then sequenced on an Illumina NovaSeq 6000 (Illumina, San Diego, USA) by Gene Denovo Biotechnology Co., Ltd. (Guangzhou, China).

### Data processing

Data preprocessing was carried out using Seurat V4.1.0 and Space Range 1.2.1 (10×Genomics) [30]. High-resolution H&E staining histology images and raw sequencing reads were entered into the Spaceranger Count pipeline to create feature barcode matrices that captured the spatiality of the data, which were used for downstream analysis. During this process, reads were spliced-aware aligned to the human reference genome (GRCh38), and tissues and fiducial spots were automatically detected and aligned. To minimize interference from the urethra, spots around regions exhibiting elevated expression of Uroplakin 3A (UPK3A) were excluded.

Following normalization with SCTransform, runPCA (nPCs=50) was applied to the V22W-5 and V22W-6 datasets for principal component analysis (PCA). The datasets were then merged using anchor-based canonical correlation analysis (CCA) integration, with the normalization method “SCT” employed to mitigate batch effects. Subsequently, dimensionality reduction, clustering, and visualization were completed using the functions FindNeighbors (dims=1:50), FindClusters (resolution=0.6), and RunUMAP (dims=1:50).

Differential expression analysis was conducted to identify spatially variable genes within all clusters and specifically within the epithelium utilizing the FindMarkers function in the R package Seurat. The ‘test.use = wilcox’ parameter was applied, employing a Wilcoxon rank sum test to identify differentially expressed genes (DEGs) between two groups of cells. In our vaginal spatial transcriptomics analysis, the expression value of each gene in a given region or cluster was compared against the rest of the spots using the Wilcoxon rank sum test. The selection criteria for DEGs included an adjusted *p* value <0.05 and a log _2_(fold change)>1.0 (representing at least a 2-fold difference), guiding subsequent Gene Ontology (GO) analysis [ 31, 32] . For a more focused exploration within the epithelium, we identified DEGs between the four epithelial zones. Genes meeting the criteria of an adjusted *p* value <0.05 and a log _2_(fold change)>0.585 (equivalent to at least a 1.5-fold difference) were then used to investigate KEGG pathways ( https://www.kegg.jp). All enriched pathways within each epithelial zone were visualized through the creation of bubble plots for visualization.

### Histology and immunostaining

Fresh fetal vaginal tissue samples were fixed in 4% paraformaldehyde for 24‒48 h at 4°C, followed by paraffin embedding after dehydration and hyalinization. The paraffin-embedded tissues were cut into 5-μm thick slices and stained with Masson’s trichrome (BP028; BASMEDTSCI, Wuhan, China) according to the kit instructions.

For tissue fixation and H&E staining, the tissue sections on Visium Slides were first fixed in methanol (Sigma, St Louis, USA) at -20°C for 30 min, followed by H&E staining. After fixation, sections were incubated in isopropanol (Sigma) for 1 min, air-dried, stained with hematoxylin (Agilent Technologies) for 7 min, rinsed with ddH _2_O, treated with Bluing buffer (Agilent Technologies) for 2 min, rinsed again with ddH _2_O, incubated with Eosin Mix (Sigma) for 1 min at room temperature, and then washed. Finally, slides were incubated at 37°C for 5 min. Following the staining process, tissue section images were acquired for sample checking and subsequent data analysis.

In our immunohistochemical (IHC) staining protocol, tissue slices were dewaxed, rehydrated, and subjected to the elimination of endogenous peroxidase with 3% hydrogen peroxide. Subsequently, they were subjected to heat-mediated antigen retrieval and peroxidase blocking using 2% bovine serum albumin (BSA; Biofroxx, Einhausen, Germany) for 30 min at room temperature. After being blocked with 2% BSA, the sections were incubated with primary antibodies overnight at 4°C, followed by incubation with HRP-conjugated secondary antibodies at room temperature for 8 min. Antigen signals were visualized using 3’3-diaminobenzidine, and sections were counterstained with hematoxylin before sealing and examination with a microscope (DMi8; Leica, Wetzlar, Germany).

For immunofluorescence (IF) staining, the steps preceding IF primary antibody incubation were identical to those preceding immunohistochemical antibody incubation. Tissue slices were incubated with primary antibodies overnight at 4°C and then with secondary antibodies at room temperature for 1 h in the dark. The nuclei were labelled with DAPI (Abcam, Cambridge, USA). Fluorescence images were captured using a fluorescence microscope (DMi8; Leica).

Both protocols employed a variety of antibodies from different suppliers, as detailed in Supplementary Table S1.

To assist in validating the spatial characteristics of certain molecules, we adapted IHC staining images from the Human Protein Atlas ( proteinatlas.org) [33]. These images are available under the Creative Commons Attribution-ShareAlike 3.0 International License, allowing for free adaptation, including remix, transform, and build upon the material for any purpose, even commercially. These include IHC staining of SPINK7 (human protein atlas, https://www.proteinatlas.org/ENSG00000145879-SPINK7/tissue/vagina#img), C15orf48 (human protein atlas, https://www.proteinatlas.org/ENSG00000166920-C15orf48/tissue/vagina#img), PI3 (human protein atlas, https://www.proteinatlas.org/ENSG00000124102-PI3/tissue/Vagina#img), IGSF9 (human protein atlas, https://www.proteinatlas.org/ENSG00000085552-IGSF9/tissue/vagina#img) and TP73 (human protein atlas, https://www.proteinatlas.org/ENSG00000078900-TP73/tissue/vagina#img).

## Results

### Spatial RNA sequencing of the human fetal vagina

**Figure 1:**
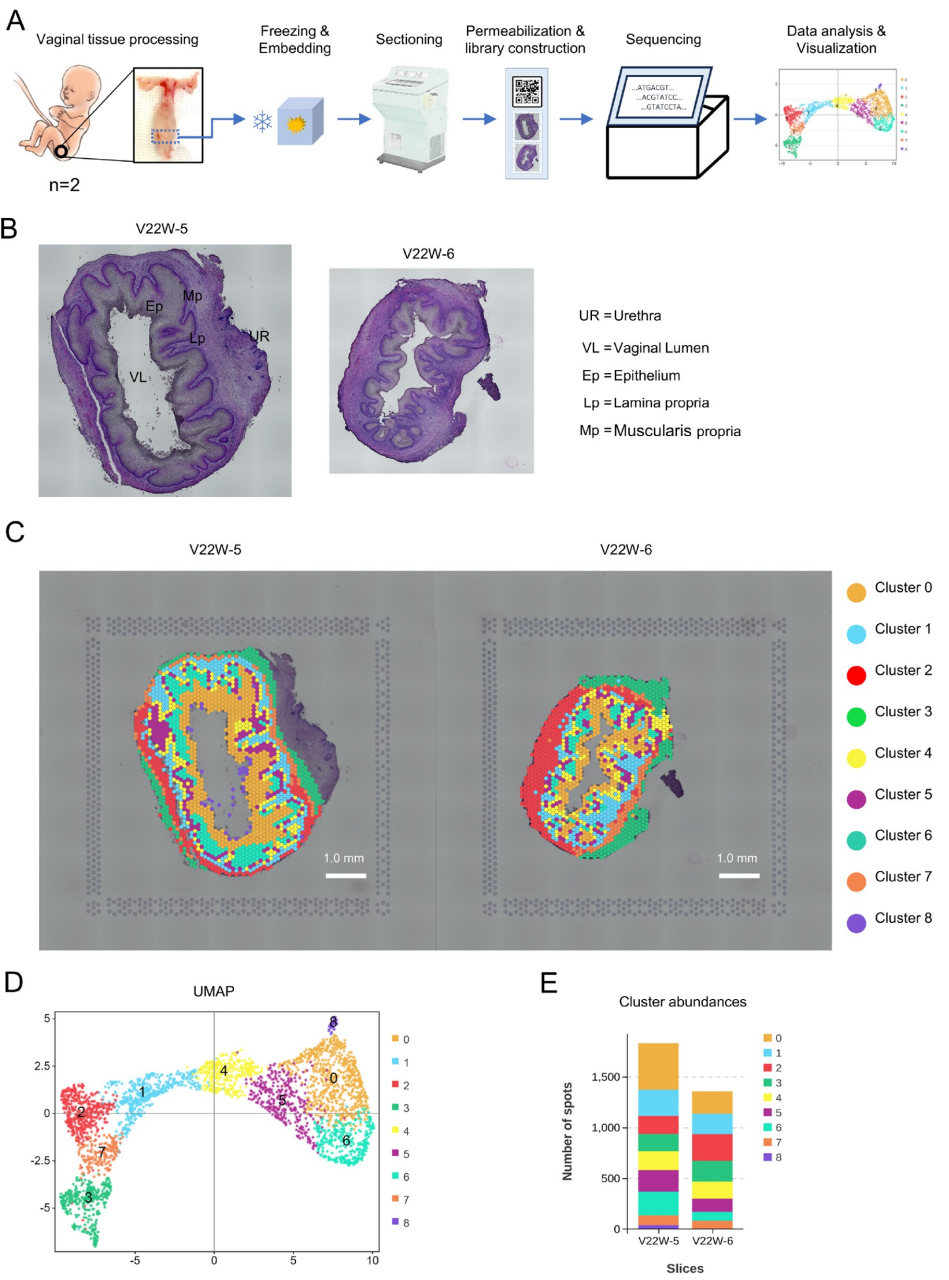
Figure 1 Spatial transcriptome analysis of the human fetal vagina (A) Spatial transcriptomic analysis workflow of the human fetal inferior/lower vagina. (B) H&E-stained cross-sections of vaginas from fetuses at 22 weeks of age near the orifice or introitus (left: V22W-5; right: V22W-6). (C) Spatial transcriptomics results mapped onto the two vaginal sections (left: V22W-5; right: V22W-6). (D) UMAP clustering of cell-covered spots from two tissue sections, resulting in 9 unique clusters; spots within the same cluster were color-coded identically. (E) Cluster abundance analysis of V22W-5 and V22W-6 based on spot numbers.

To explore the molecular features of the human vaginal epithelium, we employed a 10×Genomics Visium spatial gene expression assay. This involved the analysis of tissues derived from two human fetuses (22 ^+5^ weeks and 22 ^+6^ weeks), designated V22W-5 and V22W-6, respectively ( Figure 1A). Both tissue sections, obtained from cross-sections of the fetal vagina near the vaginal orifice, underwent optimal permeabilization ( Supplementary Figure S1B) before library construction and sequencing. H&E staining revealed distinct boundaries between the lamina propria (LP) and layered epithelia, although the cellular lamina propria exhibited poor demarcation from the muscularis propria (MP) ( Figure 1B). The observed structure closely mirrors that of the vaginal epithelia of reproductive-aged women, aligning with previous findings indicating well-developed vaginal epithelia in 20-week-old fetuses [ 7, 23] .

To ensure data integrity, we took precautions to minimize interference from the urethra, which can displace the muscularis propria along the anterior wall of the vagina ( Figure 1B, UR). Specifically, we excluded spots exhibiting elevated expression of the urothelium marker Uroplakin 3A (UPK3A) [6] and their adjacent regions ( Figure 1B,C, and Supplementary Figure S1C). Additionally, spots located outside the vaginal wall were also excluded from the analysis ( Figure 1B,C). The resulting dataset encompassed 3187 spots, with 1831 from V22W-5 and 1356 from V22W-6. The median number of genes per spot was 4337 for V22W-5 and 6032 for V22W-6. Each spot is expected to encompass approximately 1‒10 total cells, depending on their composition. Overall, 24,253 genes were identified in the V22W-5 vaginal section, and 25,400 genes were identified in the V22W-6 vaginal section.

After normalization, integration, and dimensionality reduction, the spots were clustered at a resolution of 0.6, which yielded clusters closely aligned with the anatomical features of the vagina. The uniform manifold approximation and projection (UMAP) plot containing spots from the two vaginal sections revealed 9 distinct clusters ( Figure 1D,E) and illustrated their relative spatial relationships. Spots within the same cluster that shared similar transcriptomes were labelled with the same color, maintaining consistency across both vaginal sections ( Figure 1E). Based on the morphological and spatial location within the slices ( Figure 1C), we assigned the 9 clusters to specific regions: epithelia (clusters 4, 5, 6, 0, and 8), lamina propria (cluster 1), muscularis propria (clusters 2 and 7) and cluster 3. We designated these clusters as the shed vaginal epithelial cells (cluster 8), superficial epithelium zone (superficial Z, cluster 0), intermediate epithelium zone (intermediate Z, cluster 6), parabasal epithelium zone (parabasal Z, cluster 5), basal epithelium zone (basal Z, cluster 4), lamina propria (cluster 1), ventral muscularis propria (cluster 7) located in the anterior vaginal wall, dorsal muscularis propria (cluster 2) situated in the posterior vaginal wall, and cluster 3. As the histological region of cluster 3 remains unknown and is not the focus of our study, we refrain from naming it and continue to refer to it as cluster 3. The identification of distinct regions and spatial patterns in the distribution of diverse cell types throughout the vaginal wall, especially within the epithelium, suggests the existence of unique cellular subpopulations with potentially specific functions. Furthermore, the observed asymmetric allocation of clusters, particularly evident in the anterior and posterior vaginal walls, underscores significant location-dependent heterogeneity within the tissue. This heterogeneity, which is characterized by variations in cell type composition across clusters, points to distinct microenvironments within the vaginal wall, thereby enhancing our nuanced comprehension of its cellular landscape.

### Spatially variable features and cluster-specific molecular markers revealed by differentially expressed genes in the fetal vagina

**Figure 2:**
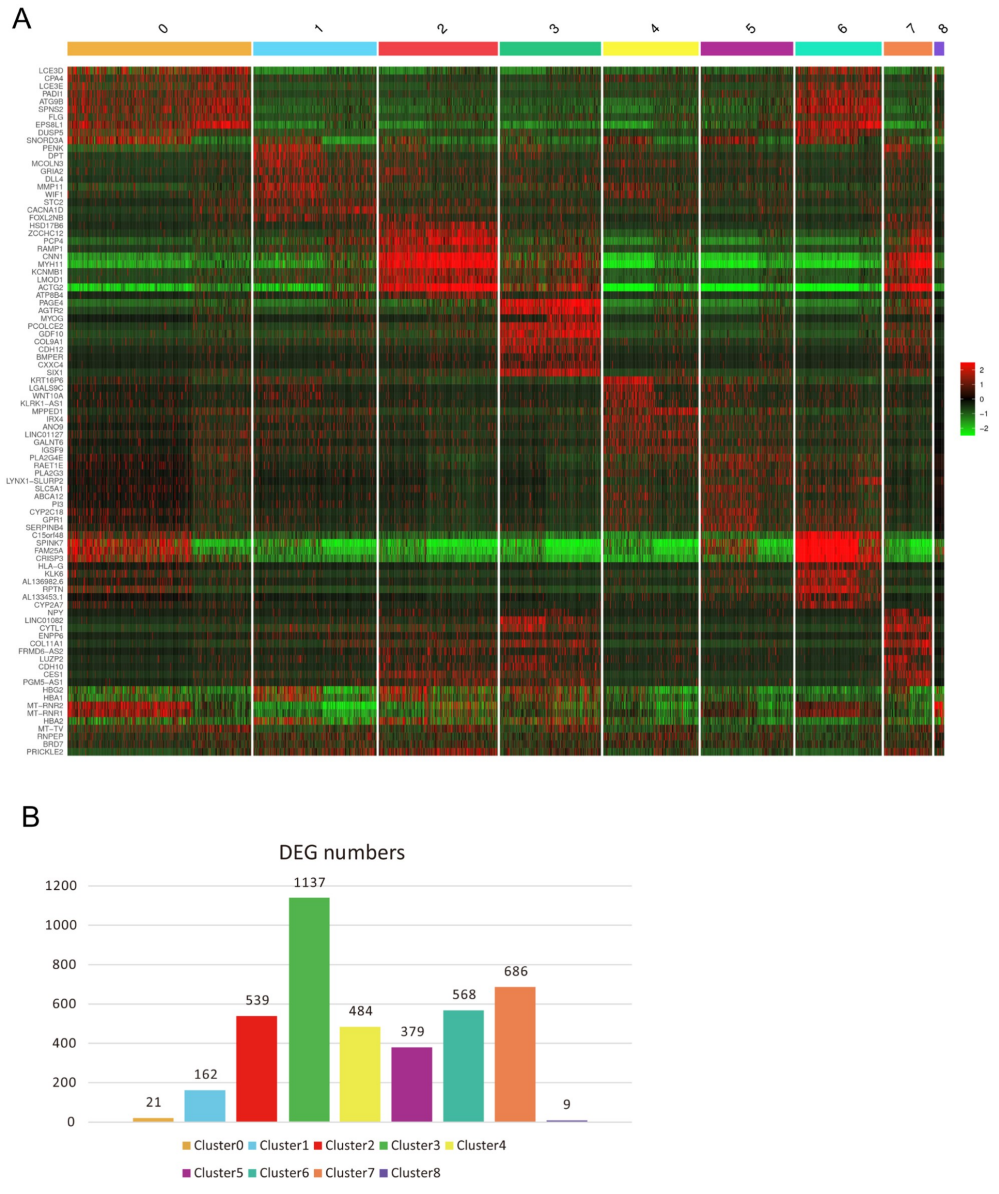
Figure 2 Spatially variable features of the human fetal vagina revealed by differential gene expression analysis (A) Heatmap presenting the top 10 DEGs per cluster, sorted by log 2(fold change). For clusters with fewer than 10 genes, all DEGs are displayed. (B) Variability in the number of DEGs across different clusters.

The identification of distinct regions and spatial distribution of diverse cell types across the vaginal wall suggest unique functions. Spatially variable features in molecular profiles enable the capture of nuanced characteristics within these clusters. To achieve this goal, we first identified the DEGs and analyzed the top 10 DEGs ( Figure 2A).

**Figure 3:**
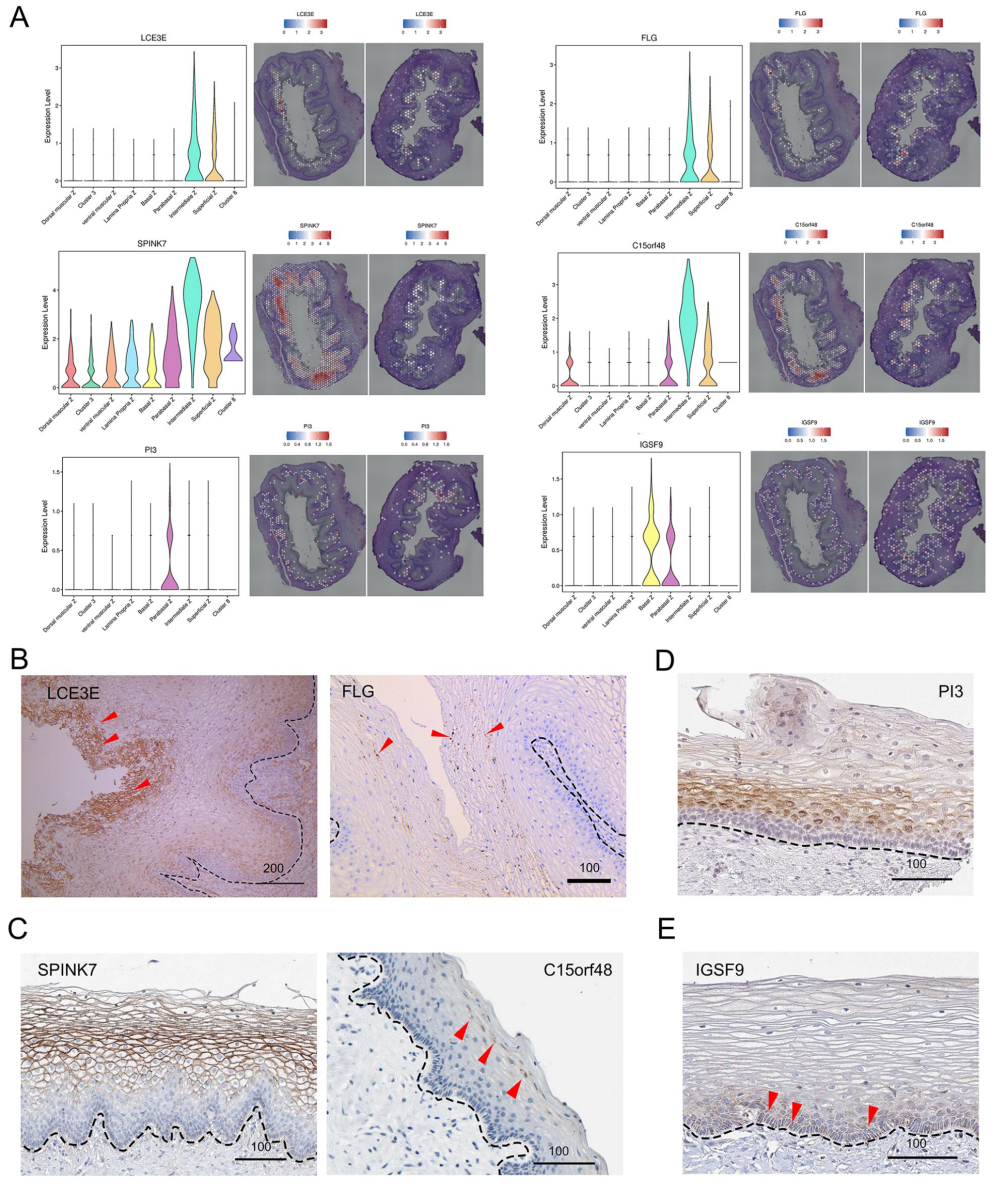
Figure 3 Zone-specific marker expression in the human fetal vaginal epithelium (A) Left: violin plot depicting the spatial distribution of DEGs, including LCE3E, FLG, SPINK7, C15orf48, PI3, and IGSF9. Right: spatial mapping further illustrates the specific zones where these genes are predominantly expressed. (B) IHC staining of LCE3E (red triangle arrow) and FLG (red triangle arrow) in fetus vagina. (C) IHC staining of SPINK7 and C15orf48 (red triangle arrow) in the adult vagina. (D) IHC staining of PI3 in the adult vagina. Notably, there are PI3-negative cells in the basal layer. (E) IHC staining of IGSF9 in the adult vagina. Scale bars are provided in the panels, with units in micrometers (μm). Notably, SPINK7, C15orf48, PI3, and IGSF9 data were cross-validated using the Human Protein Atlas (HPA) for additional confirmation (see Methods for details). Scale bars are shown in μm.

Among the nine clusters, the superficial zone (cluster 0) exhibited the lowest number of DEGs, with only 21 identified, in comparison to the other epithelial regions, except for the shed cells ( Figure 2B). Cluster 0 encompasses the superficial layers of the vaginal epithelium and serves as the interface between the host and the environment. Within this specific zone, late cornified envelope 3D (LCE3D), late cornified envelope 3E (LCE3E), and filaggrin (FLG) exhibited elevated expressions ( Figures 2A and 3A), which is indicative of terminally differentiated cells in the squamous epithelium of the epidermis [ 34, 35] . Moreover, IHC staining of LCE3E and FLG in fetal samples revealed predominant expressions in the superficial layers ( Figure 3B). This observation supports the notion that the superficial zones of the vaginal epithelium comprise terminally differentiated cells that undergo keratinization in the squamous epithelium and are poised for shedding [ 12, 13] . Moreover, our IHC staining of FLGs aligned with previous reports on adult women ( https://www.proteinatlas.org/ENSG00000143631-FLG/tissue), suggesting that at 22 weeks, the vaginal epithelium is mature in terms of squamous differentiation and already exhibits similarities to that of adult women. Furthermore, these DEGs in the superficial zone also displayed high expression levels in the intermediate epithelial zone (cluster 6) ( Figures 2A and 3A), implying that these populations share certain characteristics and suggesting a gradual reduction in transcript levels from intermediate to superficial cells.

In the intermediate zone (cluster 6), the expression levels of SPINK7 (serine peptidase inhibitor, kazal type 7) and C15orf48 were significantly greater than those in the other clusters ( Figures 2A and 3A). This spatial expression feature was consistently observed via IHC staining of samples from adult women ( Figure 3C), confirming the candidacy of these proteins as markers for cells in the intermediate zone. SPINK7 is known to regulate differentiation, barrier function, and inflammatory responses in the esophageal squamous epithelium [36], while C15orf48 plays a role in cytoprotection and the modulation of immune responses during inflammation [37]. These findings collectively imply that the intermediate zone may play a role in supporting immunization and pathogen defense, contributing to the maintenance of vaginal homeostasis [ 16, 35‒ 38] .

In the parabasal zone (cluster 5), PI3 (peptidase inhibitor 3) could mark these cells well. Our sequencing results also showed that its RNA expression was highest in the parabasal region of the vaginal epithelium ( Figures 2A and 3A), which is consistent with the IHC staining results in adult samples ( Figure 3D). Moreover, our data showed that IGSF9 (immunoglobulin superfamily member 9) strongly labelled the basal zone ( Figures 2A and 3A), which is similar to that in the adult vagina ( Figure 3E).

Cluster 1, identified as the lamina propria, provides a unique microenvironment crucial for vaginal epithelial proliferation, differentiation and maintenance [ 9, 12, 38– 42] . It is distinguished from the vaginal epithelium by a delicate basement membrane primarily composed of extracellular matrix (ECM) [8]. The predominant genes whose expressions are upregulated in this zone are associated with ECM, including metallophosphoesterase domain containing 1 ( *MPPED1*), matrix metallopeptidase 11 ( *MMP11*), stanniocalcin 2 ( *STC2*), and delta-like canonical notch ligand 4 ( *DLL4*) ( Figure 2A and Supplementary Figure S2A). Masson staining revealed abundant blue-stained collagen fibers in the subepithelial region, indicating a dense extracellular matrix ( Supplementary Figure S2B). The ECM serves as a resilient scaffold for vaginal cells, promoting adhesion, proliferation, and migration [39]. Gene Ontology (GO) analysis of the DEGs indicated a notable angiogenic tendency ( Supplementary Figure S2C). Immunohistochemical staining confirmed angiogenesis, as indicated by the presence of CD31, CD34, and VWF (von Willebrand factor) [ 40, 41] ( Supplementary Figure S2D). This finding aligns with prior studies highlighting the rich vascular network complexity of the vagina [41]. Notably, VWF expression was detected in both the basal and parabasal layers of the vaginal epithelium ( Supplementary Figure S2D).

Clusters 2, 3, and 7 exhibited a shared upregulation of genes related to muscle cells, including ACTG2 (smooth muscle marker actin gamma 2), CNN1 (calponin 1), MYH11 (myosin heavy chain 11), and PCP4 (Purkinje cell protein 4) ( Figures 2A and S3A). Additionally, NPY (neuropeptide Y) was uniquely expressed in cluster 7, corresponding to the muscularis propria located in the anterior wall of the vagina ( Figure 2A). This discovery drew our attention to the nervous system in the vagina, which is responsive to both mechanical and chemical cues, regulates functions such as blood flow, lubrication, and tissue integrity, and plays a crucial role in reflexes related to parturition [43]. Our assessment of neural-related marker molecules, such as vasoactive intestinal polypeptide (VIP), pan-neuronal marker PGP9.5 (UCHL1), nerve growth factor receptor (NGFR), and S100 calcium-binding protein B (S100B), revealed their predominant expressions in the muscularis propria (clusters 2 and 7) and cluster 3 ( Figure 2A and Supplementary Figure S3B).

### Cross-validation of cluster assignment through cell type-specific markers and histological characteristics

**Figure 4:**
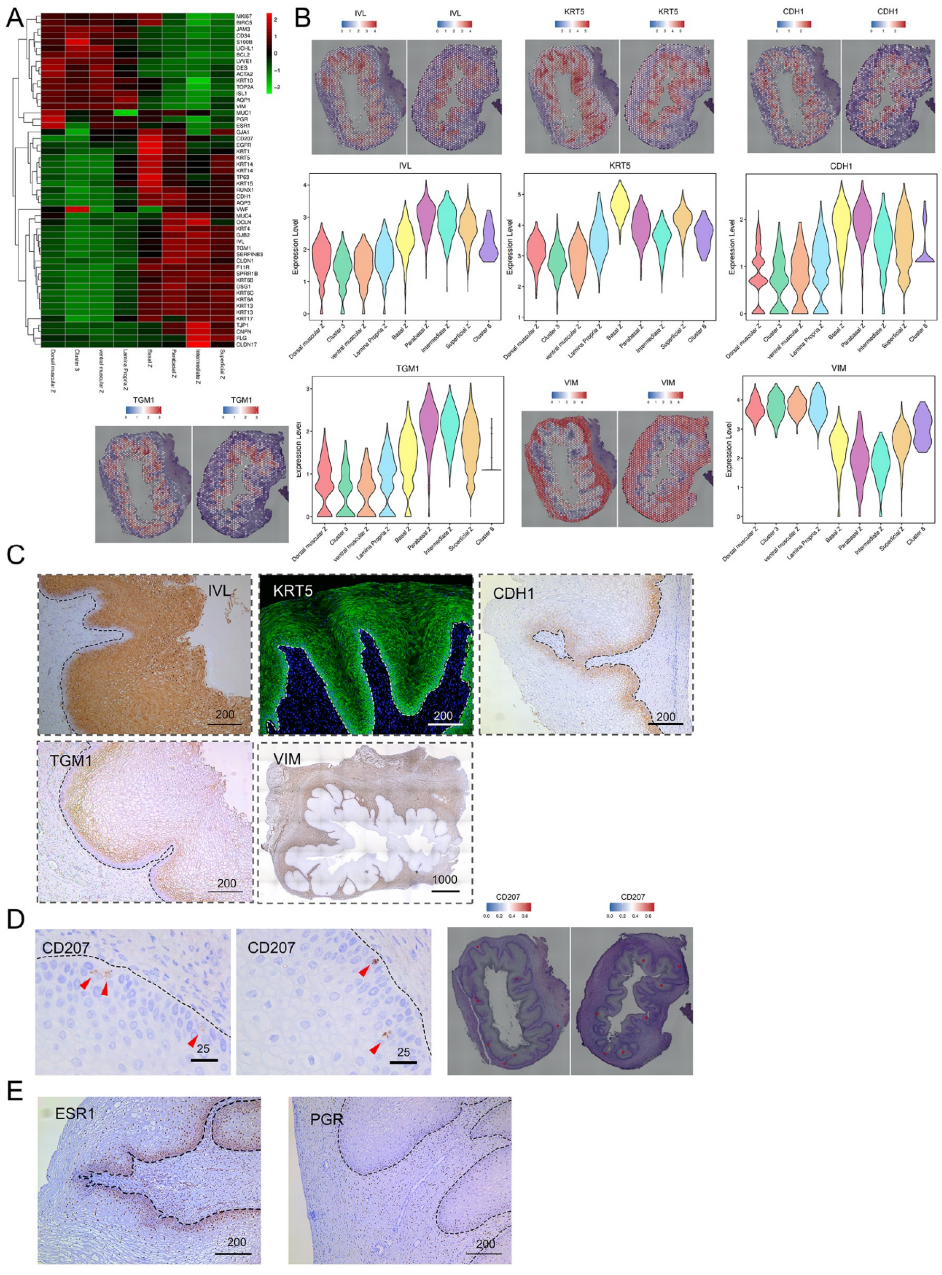
Figure 4 Cluster assignment using cell type-specific markers and constraints from histological characteristics (A) Heatmap displaying the relative expression levels of canonical markers in each cluster. (B) Spatial distribution features of markers (IVL, KRT5, CDH1, TGM1, and VIM) in the human fetal vagina illustrated through spatial mapping and violin plots. (C) Verification of markers expressed in vaginal epithelia by immunofluorescence (KRT5) and immunohistochemical staining (IVL, CDH1, TGM1, and VIM) in the fetal vagina. (D) IHC staining (left, middle) and spatial mapping of CD207 in the basal layer (red triangular arrow) in the fetal vagina. (E) IHC staining of PGR and ESR1 in the human fetal vagina. Scale bars are shown in μm.

Furthermore, we integrated key markers identified in previous studies into our analysis, mapping them onto our spatial transcription data. We organized these markers, which are indicative of distinct vaginal characteristics, into a heatmap for better visualization ( Figure 4A). Additionally, spatial characteristics observed on the H&E section slices were also incorporated as constraints to guide the cluster assignment process ( Supplementary Figure S4A). In the majority of cases, we found that well-established cell type markers reliably identified specific cell zones within vaginal tissue. For instance, AQP1 marks the subepithelial area, aligning with capillaries and venules, as reported previously [44]. AQP3 was observed in the epithelial zone, consistent with its known role as an epithelial marker [44]. Similarly, ACTA2 [45] and Desmin (DES) [46] in the muscularis propria confirmed their utility in identifying smooth muscle cells. Additional markers, including ISL1 for the vaginal mesenchyme [6], LYVE1 for lymphatic vessels [47], Tp63 for basal region cells of the vaginal epithelium [6], and F11R for the parabasal and basal layers of the epithelium [8], were observed in corresponding locations with related patterns ( Figure 4A and Supplementary Figure S4A). Overall, these findings highlight the robustness and precision of these markers in characterizing distinct cellular components within vaginal tissue, further confirming our assignment of clusters to corresponding zones of the vagina.

To further validate the applicability of the markers chosen for assigning clusters in the vaginal tissue of 22 ^+^-week-old fetuses, we conducted IF and IHC experiments for selected markers. Involucrin (IVL), a subunit of keratinocyte cross-linked envelopes, is a distinctive marker for suprabasal differentiation in stratified squamous epithelium [48]. IVL was highly expressed in the parabasal region of the vaginal epithelium ( Figure 4B, adjusted *P* value<0.0001). This protein appears in the cells immediately above the basal layer ( Figure 4C). At 20 weeks of gestation, the expression of basal cytokeratin 5 (KRT5) increases and is crucial for maintaining the integrity of the squamous epithelium [49]. In our study, KRT5 expression was highest in the basal region ( Figure 4B). IF revealed intense expression of KRT5 in the fetal vaginal epithelium at 22 ^+^ weeks, particularly in the basal zone ( Figure 4C). The IF staining pattern aligned with the trend of RNA expression obtained from sequencing data. CDH1 (E-cadherin) is the common transcellular component of all epithelial adherens junctions, and it is highly expressed in the parabasal and basal layers of the vaginal epithelium in adult women [8]. Consistent with these findings, our study showed that the CDH1 gene had the highest expression in both parabasal and basal regions ( Figure 4B). Moreover, the protein expression of CDH1 was notably strong in the basal and parabasal layers ( Figure 4C). Transglutaminase 1 (TGM1), which is regulated by estradiol, plays a key role in the terminal differentiation of rat vaginal epithelial cells [50]. Our sequencing results also showed that its expression was highest in the parabasal region of the vaginal epithelium, which is consistent with the IHC staining results ( Figure 4B,C). Additionally, the wide expression of the stromal cell marker VIM (Vimentin) [51] is notable in nonepithelial areas, and IHC staining for VIM can effectively distinguish between epithelia and mesenchyme ( Figure 4C). Taken together, these results suggest that our sequencing results are reliable, our clustering strategy is reasonable, and the gene expression pattern in the vaginal epithelium of 22-week-old foetuses closely resembles that of adults.

Since the vaginal epithelium plays an important role as an immune barrier, we also examined the expressions of several immune cell markers in the sequencing data. Specifically, we observed the expression of CD68 in macrophages [52], which was primarily expressed in the intermediate zone ( Supplementary Figure S4B). Additionally, CD207, a marker for Langerhans cells [53], was expressed in the basal region ( Figure 4D). Furthermore, CD45, which is involved in the initiation of T-cell receptor signaling [54], was primarily expressed in the basal zone ( Supplementary Figure S4C). These results suggest that immune cells are already present in the vaginal epithelium of 22 ^+^ week-old fetuses and that the expression of immune cells in the vaginal epithelium is location-specific.

The estrogen-stimulated vaginal epithelium expressed ESR1 widely in the vaginal mesenchyme and epithelium ( Figure 4E). In our fetal samples, progesterone receptor (PGR) was uniformly expressed in the epithelium of the basal region and widely expressed in the subepithelial region ( Figure 4E), suggesting that steroid hormones broadly influence the development of the vagina at this time.

### Keratin genes reveal the heterogeneity of vaginal epithelial cells

**Figure 5:**
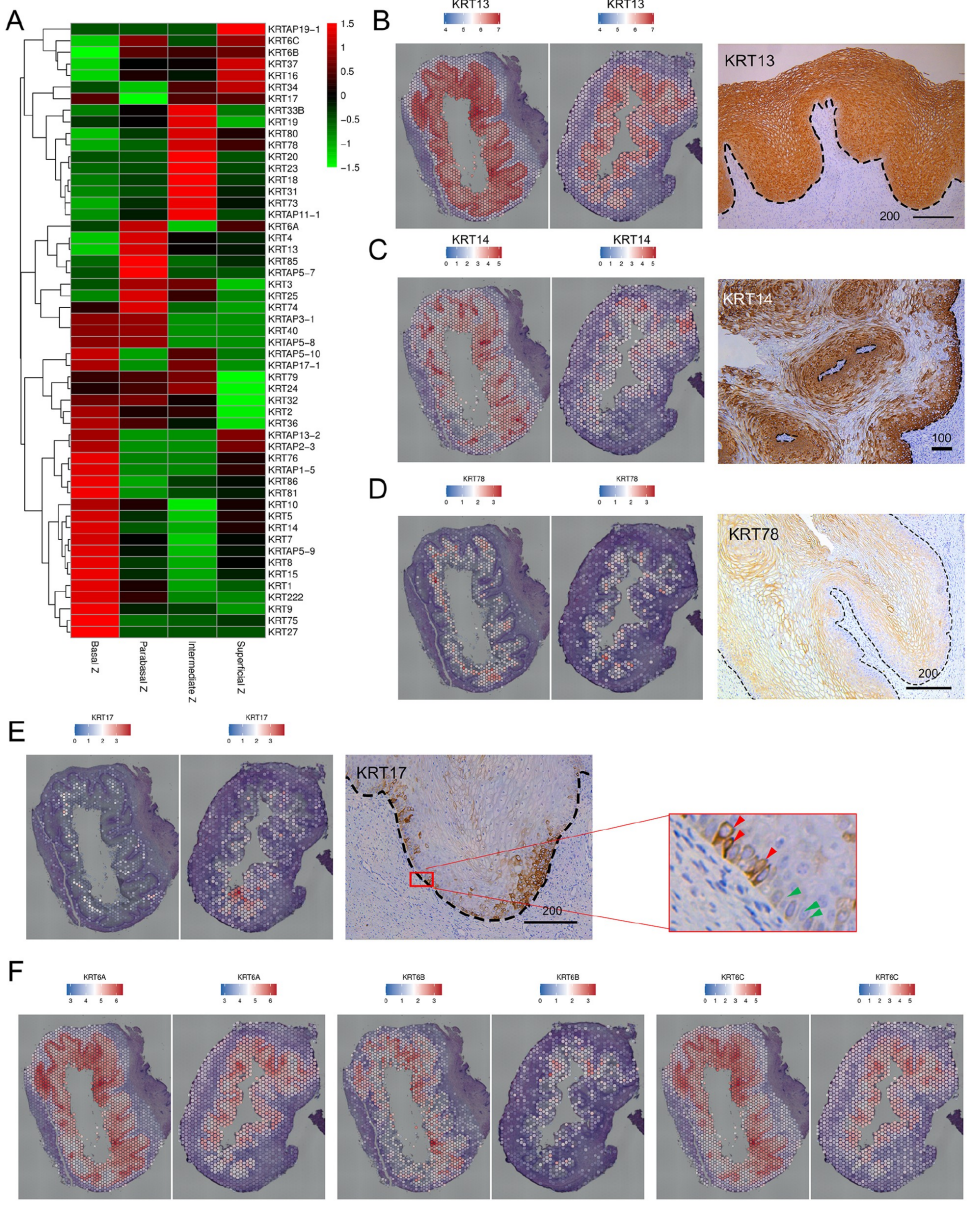
Figure 5 Keratin gene analysis reveals heterogeneity of the vaginal epithelium (A) Heatmap analysis illustrating the zone-specific dominant keratin family genes across the vaginal epithelium. (B) Left: spatial mapping of KRT13 across the vaginal epithelium. Right: IHC staining of KRT13 in fetal vagina. (C) Spatial mapping and IHC staining of KRT14 in fetal vagina. (D) Spatial mapping of KRT78 and IHC staining of KRT78 in fetal vagina. (E) Spatial mapping of KRT17 and IHC staining of KRT17 in fetal vagina. KRT17 positivity (red arrow); KRT17 negativity (green arrow). (F) Spatial mapping of KRT6A, KRT6B, and KRT6C. Scale bars are shown in μm.

The vaginal epithelium, characterized by a diverse array of molecular markers ( Figure 4A), demands a targeted examination of pivotal molecules to elucidate their interactions and developmental trajectories. Leveraging insights from studies on keratin expression in the human epithelium, especially its pivotal role in differentiation and regulation [55], we analyzed keratin proteins (KRTs) within the vaginal epithelium ( Figure 5A and Supplementary Table S2) to elucidate the underlying processes involved. Through our comprehensive spatial transcriptomics analysis of the vagina, we were able to identify a substantial representation of human genomic keratins within the epithelium. Specifically, we detected 42 out of the 56 reported keratins, accounting for 75% ( Supplementary Table S2). Among these, the most abundantly expressed keratins in the epithelium included KRT13, KRT6A, KRT5, KRT14, KRT4, KRT6C and KRT19 ( Supplementary Table S2). To unravel the intricacies of epithelial heterogeneity with respect to proliferation and differentiation, we systematically clustered the detected keratins and visualized them in a heatmap ( Figure 5A). This analysis provided valuable insights into the specific keratins associated with each zone of the epithelium.

Our findings revealed distinct expression patterns of keratins across different zones, highlighting spatial heterogeneities. First, KRT13 was strongly expressed across all epithelial zones ( Figure 5B), emphasizing its distinct and potentially crucial role in shaping the molecular landscape of the vaginal epithelium. Notably, several KRTs were predominantly expressed in the basal region ( Figure 5A). In particular, KRT5/14, which was most abundant in the basal zone, exhibited a gradual decrease in expression towards the parabasal and intermediate zones ( Figures 4C and 5C), consistent with tissue stratification. Interestingly, a marked increase in KRT5/14 expression was observed in the superficial zone, prompting further exploration of its functional significance ( Figure 5A). In contrast to KRT5/14, KRT1/10 demonstrated relatively lower levels across all zones ( Supplementary Table S2). KRT78 was predominantly expressed in the intermediate and superficial regions. This finding was confirmed by heatmap analysis and spatial mapping and further substantiated by IHC, which revealed an ascending pattern from the basal to the intermediate region ( Figure 5A,D). Interestingly, KRT17 forms discrete clusters confined to a small region ( Figure 5E). In the basal layer, KRT17 was observed in only a subset of cells ( Figure 5E, enlarged, red), while other cells lack KRT17 expression ( Figure 5E, enlarged, green), revealing heterogeneity within the basal layer. Furthermore, our analysis focused on the three isotypes of KRT6, which, along with KRT16/17, play a crucial role in epithelial healing [56]. These isotypes exhibited wide distribution patterns along the vaginal epithelium ( Figure 5F).

### Functional enrichment analysis of DEGs among vaginal epithelial zones

**Figure 6:**
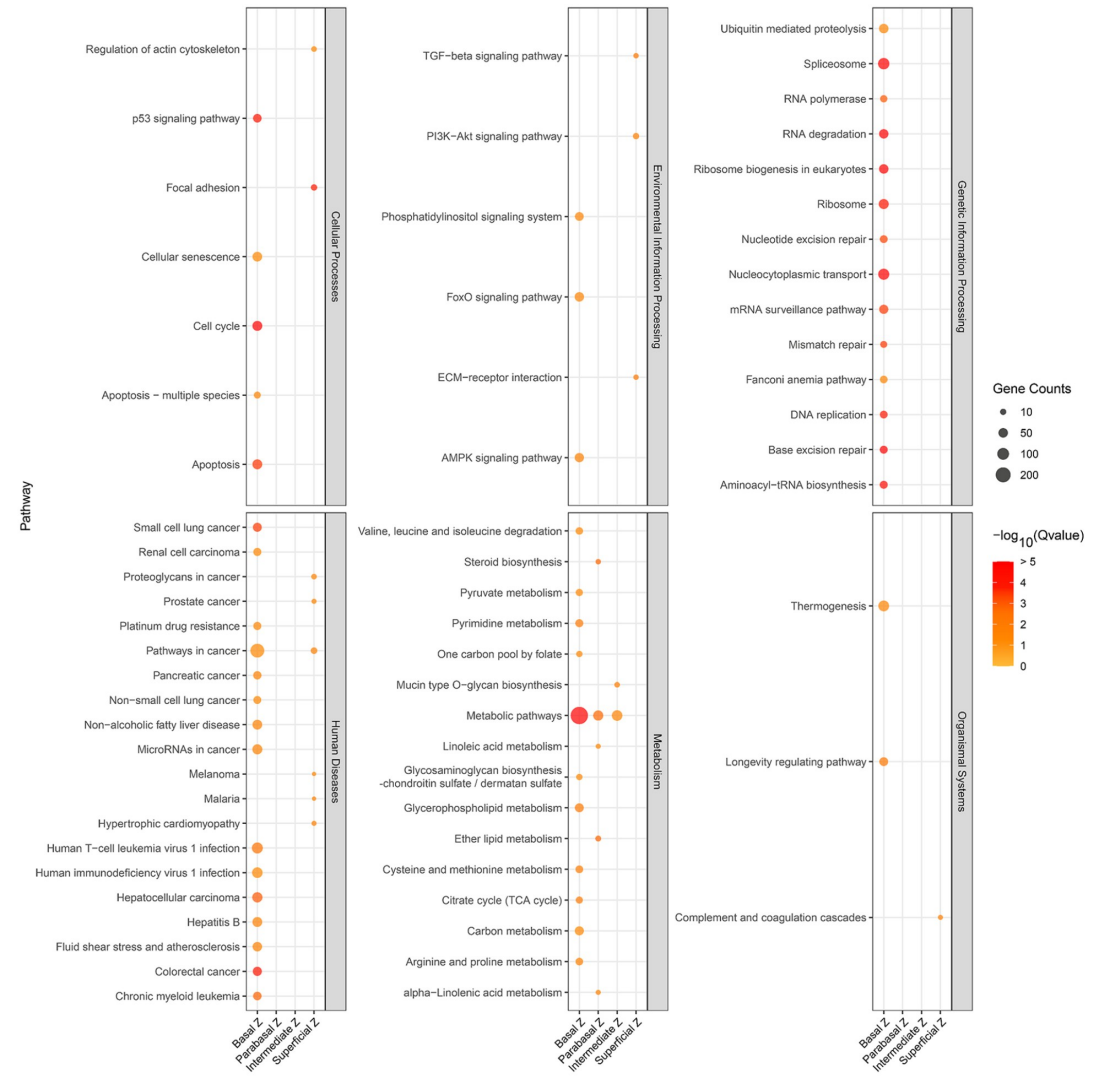
Figure 6 KEGG analysis reveals diverse pathways associated with various epithelial zones Bubble plot presenting all the KEGG pathways to elucidate the pathways active within each region of the vaginal epithelium. The colors represent the ‒log 10(q-value), and the bubble size indicates the number of genes enriched within each pathway.

Given our observation of distinct gene expression patterns in various regions of the vaginal epithelium, the functions and pathways of these epithelial regions were enriched ( Figure 6) according to KEGG analysis. This comprehensive analysis allowed us to delve more deeply into the biological roles played by the DEGs ( Supplementary Figure S5A).

Notably, the DEGs in the basal cell zone are predominantly linked to the cell cycle, apoptosis, cellular senescence, p53 signaling pathway, citrate cycle (TCA cycle), and metabolic pathways ( Figure 6). Additionally, these genes were found to be associated with genetic information processing pathways such as the nucleocytoplasmic transport, DNA replication, nucleotide excision repair, mismatch repair, base excision repair, spliceosome, RNA polymerase, and ribosome biogenesis pathways in eukaryotes ( Figure 6). This suggests that the basal region cells of the vaginal epithelium are highly active and could play a pivotal role in the maintenance, proliferation, and differentiation of epithelial stem cells in this region.

**Figure 7:**
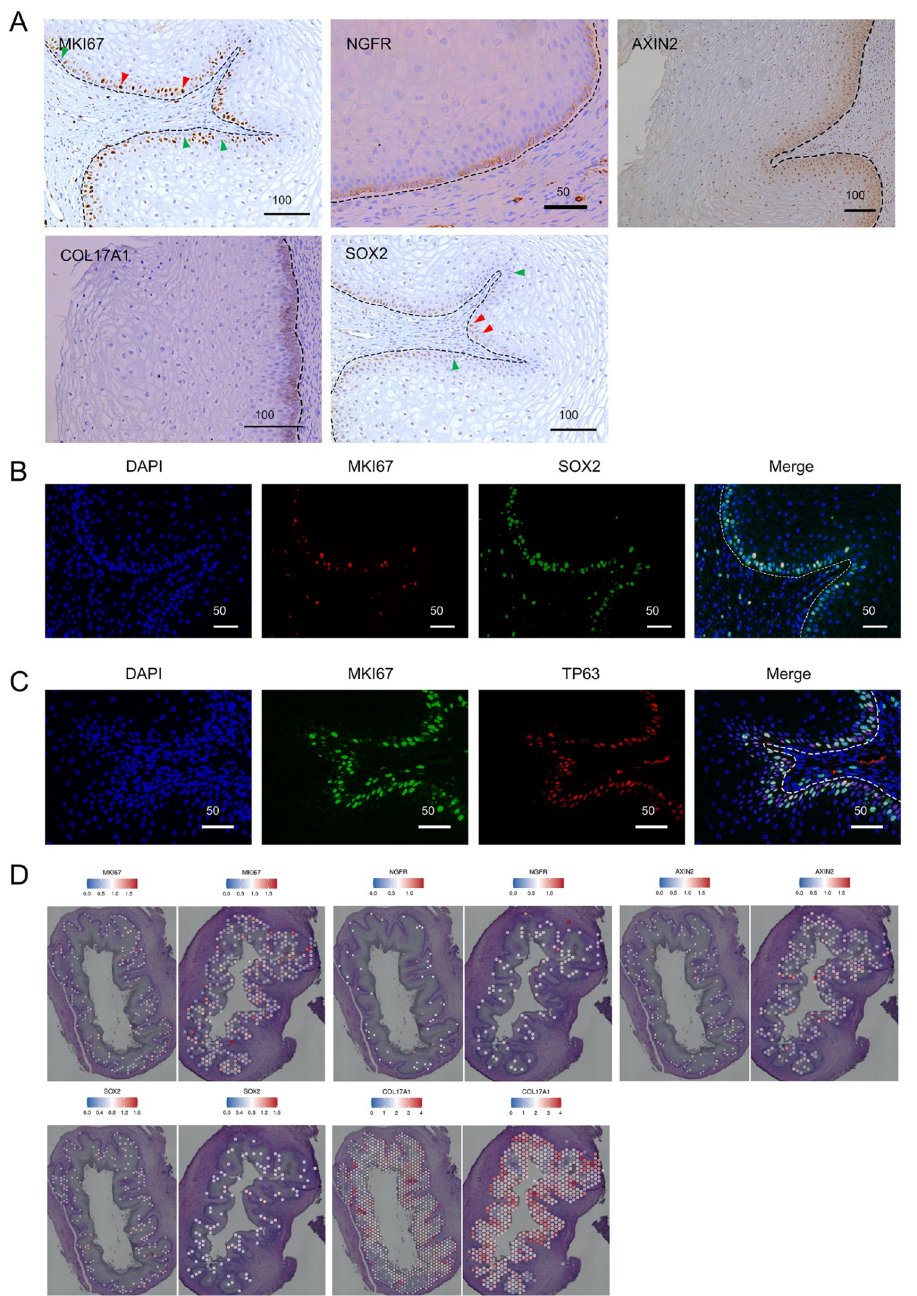
Figure 7 Potential progenitor epithelial cells in the vagina (A) IHC staining for proliferation marker (MKI67) and stem cell-related markers (NGFR, AXIN2, COL17A1, and SOX2) in the active basal layer of the fetal vaginal epithelium. (B) Immunofluorescence (IF) staining of MKI67 and SOX2 in the fetal vagina. (C) IF staining of MKI67 and TP63 in the fetal vagina. Scale bars are shown in μm. (D) Spatial mapping of MKI67, NGFR, AXIN2, SOX2 and COL17A1.

We examined several markers identified for the basal zone, along with markers typically associated with stem cells in other organs, to assess their potential as indicators of vaginal epithelial stem cells. In the vagina of 22 ^+^-week-old fetuses, proliferation was predominantly observed in the 2‒3 layers of cells in the basal region, as evidenced by MKI67 staining ( Figure 7A,D). Subsequently, we performed immunohistochemical staining for NGFR and Axin2, which are markers of mouse vaginal epithelial stem cells and are expressed in a single layer of cells in the basal region of the mouse vagina [ 12, 55] . Consistent with the spatial expression pattern observed in the mouse vaginal epithelium, NGFR labelled only one layer of cells in the basal region of the human fetal vaginal epithelium. However, AXIN2 expression was detected in several layers of cells in the basal region ( Figure 7A,D). This pattern differed from that observed in the mouse vaginal epithelium, where only a basal layer of positive cells was present.

Furthermore, we analyzed the expressions of known stem/progenitor cell markers in other organs of the fetal vagina. COL17A1, a marker of epidermal stem cells encoding a collagen with a hemibridged granule structure involved in keratinocyte adhesion [56], exhibited differential expression in the basal region of the vaginal epithelium and was specifically expressed in the basal layer ( Figure 7A,D). This expression pattern is consistent with that observed in adults (The Human Protein Atlas. https://www.proteinatlas.org/ENSG00000065618-COL17A1/tissue/vagina). Additionally, we found that SOX2, a key regulator of various stem cell types, including embryonic stem cells (ESCs) and neural progenitor cells (NPCs) [57], was predominantly expressed in the basal layer of the epithelium, although not all basal cells were SOX2 positive ( Figure 7A,D). Cells colabelled with MKI67 and subjected to SOX2 immunofluorescence were observed in the basal region ( Figure 7B). TP63, a master regulator of stratified epithelia responsible for initiating the stratification program and maintaining the self-renewal capacity of stratified epithelial stem cells [ 40, 58, 59] , is located specifically in the basal region of the vaginal epithelium. A significant overlap was observed between MKI67-positive and TP63-positive epithelial cells ( Figure 7C). TP73, a marker of basal epidermal stem cell populations located near hair follicles in scale-like skin [57], was specifically expressed in the basal region of the vaginal epithelium ( Supplementary Figure S5B,C) and was also present in the basal layers of adult vaginal samples ( Supplementary Figure S5D).

Additionally, the parabasal zone exhibited a marked increase in substance synthesis and metabolic activity. This zone was found to be abundant in various metabolic pathways, especially lipid synthesis and metabolism, including steroid biosynthesis, linoleic acid metabolism, alpha-linoleic acid metabolism, and ether lipid metabolism ( Figure 6). The intermediate zone showed an enrichment in mucin type O-glycan biosynthesis ( Figure 6). In contrast, the superficial region did not exhibit significant enrichment in metabolic pathways, which could be attributed to the terminal differentiation stage of cells in this region. Collectively, these findings underscore the high metabolic activity within the vaginal epithelium of the basal, parabasal and intermediate zones, with epithelial cells metabolizing distinct substances in specific areas.

Importantly, the basal zone also showed enrichment in diseases related to pathogenic microbial infections, such as human immunodeficiency virus 1 infection and human T-cell leukemia virus 1 infection ( Figure 6), highlighting the immune function of vaginal epithelial cells. In conclusion, our clustering strategy revealed a distinct subgroup of vaginal epithelial cells that are enriched in specific biological processes.

## Discussion

Our study employed the 10×Genomics Visium Spatial Gene Expression assay to conduct a thorough examination of cellular composition and gene expression profiles within the vaginal epithelium of two human fetuses (22 ^+5^ weeks and 22 ^+6^ weeks). This approach allowed us to determine the molecular features and heterogeneity across different epithelial layers. By incorporating spatial constraints and region-specific markers, we achieved enhanced accuracy in cluster assignments, revealing 9 distinct clusters that aligned with the anatomical features of the vagina, including the epithelial, lamina propria, and muscularis propria regions. Furthermore, the epithelium was delineated into 4 distinct regions: superficial, intermediate, parabasal, and basal zones.

In vaginal research, accurately defining layers or regions is important, as it precedes the characterization of their molecular and cellular attributes. Our study pinpointed several molecules as promising markers for distinguishing various zones. Notably, KRT13 and KRT5 were found to specifically label nearly all fetal vaginal epithelial cells, establishing them as epithelial markers ( Figures 4C and 5B). Additionally, we identified distinct markers specific to different epithelial regions: IGSF9, NGFR, COL17A1, TP63, KRT14, and TP73 consistently labelled cells in the basal region; PI3 specifically targeted parabasal cells; CDH1 marked cells in both the basal and parabasal regions; SPINK7 and C15orf48 effectively identified cells in the intermediate region; and LCE3E, FLG, and KRT78 reliably labelled cells in both the intermediate and superficial regions.

Keratin subtypes, which are essential for both structural support and metabolic process regulation, exhibit selective expression patterns in the human vagina. This heterogeneity is especially pronounced across cell types and spatial zones. Our comprehensive analysis of keratin expression patterns underscores the diversity within vaginal epithelial cells. Notably, KRT5/14 is most abundant in the basal zone and gradually decreases toward the parabasal and intermediate zones ( Figures 4C, 5A and 5C). This expression pattern parallels the regulation of epidermal stem cell proliferation, necessitating a relatively high level of KRT5/14 expression. In epidermal cells, the downregulation of K5/14 occurs as cells progress from the basal to suprabasal layers, a pattern observed in skin development [20]. This suggests that the human vaginal epithelium maintains stem cell proliferation in the basal zones while undergoing differentiation towards the superficial zone, mirroring a developmental pattern observed in the mouse vagina [12].

The vaginal epithelium, a squamous epithelium, relies on a renewal process to maintain its integrity and homeostasis. This renewal is hypothesized to involve the differentiation and proliferation of stem cells located in the basal region. Notably, the vaginal epithelium has demonstrated the capacity to fully recover from atrophy, a condition often characterized by the absence of mature differentiated keratinocytes in the upper layers of the vaginal epithelium [ 11, 58, 59] , in response to estrogen [12]. This regenerative ability suggests the presence of a population of epithelial adult stem cells within the vagina. However, despite a few studies on vaginal stem cells, no clear epithelial stem cell marker has been identified for the human vagina [12]. According to our data, the basal zone exhibits significant enrichment of pathways associated with the cell cycle, apoptosis, oxidative phosphorylation, the tricarboxylic acid (TCA) cycle, and various metabolic processes ( Figure 6). This activity suggests a potential role for this zone in the maintenance, proliferation, and differentiation of human vaginal epithelial stem cells. The transcription factor TP63 is a marker for cells in the basal region of the vaginal epithelium. Rodent studies have shown that Tp63-expressing vaginal epithelial cells have latent skin competence, explaining the occasional observation of aberrant hair follicles or sebaceous glands in the vagina [40]. In the squamocolumnar region of the anorectal junction, a population of KRT17-positive basal cells has the capacity to sustain a squamous epithelium during normal homeostasis and actively contributes to the repair of a glandular epithelium following tissue injury [60]. Similarly, in the squamocellular-columnar cervical junction, KRT17 ^-high^ P63 ^+^KRT5 ^+^ cervical cells can differentiate into both squamous and columnar cells [61]. In our vaginal samples, KRT17 was uniquely observed in a subset of cells in the basal layer ( Figure 5E), reflecting the heterogeneity of cells in this layer. Whether KRT17 is a marker for vaginal epithelial stem cells remains to be experimentally verified. The NGFR ^+^AXIN2 ^+^ marker, identified as a stem cell marker in mouse vaginal epithelia [12], labels the fetal basal layer of the human vaginal epithelium ( Figure 7A). Additionally, basal region markers such as IGSF9, COL17A1, KRT14, and TP73, along with the stem cell marker SOX2, are potential markers for human vaginal epithelial stem cells. Further research is needed to confirm the identity and function of these markers in the human vagina.

The immune function of vaginal epithelial cells is underscored by the prevalence of diseases related to microbial infections, emphasizing their crucial role as a barrier against pathogens. A particularly noteworthy finding is the spatially specific distribution of immune cells within the vagina ( Figure 4D and Supplementary Figure S4B,C), shedding light on their contribution to maintaining homeostasis across various regions. Importantly, the basal zone also showed enrichment in human immunodeficiency virus 1 infection and human T-cell leukemia virus 1 infection ( Figure 6), highlighting the immune function of vaginal epithelial cells. The parabasal zone demonstrated elevated levels of substance synthesis and metabolic activity, with a notable focus on lipids. These pathways include steroid biosynthesis, linoleic acid metabolism, alpha-linoleic acid metabolism, and ether lipid metabolism ( Figure 6). The vaginal epithelium is characterized by a high lipid composition, with phosphatidylethanolamine, cholesterol, and phosphatidylcholine reported as dominant components [60]. These lipids are believed to contribute to the epithelium’s defense against pathogenic microorganisms and regulate its permeability to water and various chemical substances. Our analysis specifically revealed enrichment in Mucin type O-glycan biosynthesis within the intermediate zone, but its biological role is unclear, and it may function as a barrier.

Our focus on vaginal samples from 22-week-old fetuses revealed striking molecular and histological parallels with those of reproductive-aged women, revealing a stratified squamous epithelium high in glycogen content [61]. However, two sequencing samples from two 22 ^+^ week fetuses are limited, and susceptibility to outcomes is influenced by individuals. To paint a more nuanced and comprehensive picture, it is imperative to extend our investigations to a wider age spectrum and more samples. This approach will yield richer temporal and spatial insights, thereby refining our understanding of the intricate functions and developmental trajectories of the vaginal epithelium. Additionally, functional validation and targeted experiments are warranted to corroborate the inferences drawn from spatial transcriptomics data.

In conclusion, our study elucidates the molecular landscape and heterogeneity of the human fetal vaginal epithelium. The integration of spatial transcriptomics, keratin profiling, and functional enrichment analyses provides a multifaceted perspective on the cellular composition and activities within distinct epithelial zones. These findings not only advance our understanding of prenatal vaginal development but also have significant implications for understanding adult epithelial characteristics and functions. These data also increase our knowledge of squamous epithelial biology. Further explorations in this area have the potential to unveil additional intricacies of vaginal tissue biology and its profound connections to health and disease.

## Supporting information

24084Supplementary_tables

24084supplementary_Figures

## Supplementary Data

Supplementary data is available at *Acta Biochimica et Biophysica Sinica* online.
