# Supplementary material for: Molecular stratification of the human fetal vaginal epithelium by spatial transcriptome analysis: Spatial transcriptome analysis of the human fetal vaginal epithelium
Source: Acta Biochim Biophys Sin (Shanghai). 2024 Apr 25;56(10):1521–36. doi: 10.3724/abbs.2024063 (PMC11612642; doi:10.3724/abbs.2024063)
Supplement: 24084Supplementary_tables [file 24084Supplementary_tables.pdf]

Supplementary Table S1. Antibodies used for immunohistochemistry (IHC) and immunofluorescence (IF)

Primary antibodies

Antigen	Supplier	Cat. #	IHC	IF
ESR1	abcam	ab23063	1:200	NA
PGR	CST	#8757	1:200	NA
CD31	abcam	ab182981	1:100	NA
CD34	abcam	ab81289	1:200	NA
KRT5	abcam	ab52635	NA	1:200
FLG	abcam	ab221155	1:1000	NA
CDH1	abcam	ab76055	1:400	NA
TGM1	proteintech	12912-3-AP	1:400	NA
CD45	abcam	ab10558	1:400	NA
VIM	abcam	ab92547	1:200	NA
KRT78	abcam	ab122578	1:200	NA
KRT17	abcam	ab51056	1:200	NA
MKI67	abcam	ab279653	NA	1:1000
MKI67	abcam	ab15580	1:200	NA
SOX2	abcam	ab97959	1:500	1:500
TP63	Millipore	ABS552	NA	1:1000
KRT5	abcam	ab52635	NA	1:200
VWF	abcam	ab6994	1:200	NA
Axin2	abcam	ab107613	1:500	NA
CK14	abcam	ab119695	1:100	NA
NGFR	abcam	ab52987	1:100	NA
COL17A1	ABclonal	A4808	1:100	NA
INVOLUCRIN	ABclonal	A13311	1:100	NA
CK13	ABclonal	A0411	1:200	NA
LCE3E	BT LAB	BT-AP03388	1:100	NA

Secondary antibodies

Antibodies	Conjugation	Dilution	Supplier	Cat. #
Goat anti-rabbit	Rhodamine	1/200	Jackson ImmunoResearh	#111-025-045
Goat anti-mouse	Rhodamine	1/200	Jackson ImmunoResearh	#115-025-003
Goat anti-rabbit	Alexa Flur 488	1/200	Jackson ImmunoResearh	#111-545-003
oat anti-mouse	Alexa Flur 488	1/200	Jackson ImmunoResearh	#115-095-003

Supplementary Table S2. Normalized expression levels of Keratin and Keratin-associated molecules in human fetal vaginal epithelium

GeneName	Basal Z	Parabasal Z	Intermediate Z	Superficial Z	Exp. Sum
KRT1	1.0452	0.6647	0.3219	0.4699	2.5017
KRT2	0.0226	0.0146	0.0156	0.0015	0.0543
KRT3	0.0198	0.0379	0.0344	0.0117	0.1038
KRT4	44.9972	109.6414	63.0063	53.022	270.6669
KRT5	109.3333	56.1458	36.2563	66.9927	268.728
KRT6A	211.2655	262.8659	179.9969	227.3994	881.5277
KRT6B	3.8616	5.9184	5.1594	5.7856	20.7249
KRT6C	41.2599	57.5102	41.4906	52.7783	193.039
KRT7	0.3729	0.2478	0.1906	0.2482	1.0595
KRT8	3.1469	1.9184	1.3125	1.928	8.3058
KRT9	0.0847	0.0583	0.0563	0.0499	0.2492
KRT10	2.8136	2.3061	1.5781	2.2643	8.9621
KRT13	575.1017	705.4752	617.475	621.2687	2519.3206
KRT14	49.8757	18.0175	13.0813	25	105.9744
KRT15	5.7571	2.6501	1.7969	2.5932	12.7973
KRT16	10.4605	10.9883	10.5188	11.1204	43.088
KRT17	2.5932	1.2799	2.2344	2.0793	8.1868
KRT18	2.596	3.0175	5.4313	3.1439	14.1887
KRT19	30.0311	30.793	37.0156	27.1248	124.9645
KRT20	0	0	0.0031	0	0.0031
KRT23	0.0141	0.0175	0.0656	0.0191	0.1163
KRT24	0.0141	0.0146	0.0156	0.0103	0.0546
KRT25	0	0.0058	0.0031	0	0.009
KRT27	0.0028	0	0	0	0.0028
KRT31	0.0254	0.0262	0.0313	0.0294	0.1123
KRT32	0.0085	0.0087	0.0063	0	0.0235
KRT33B	0.0085	0.0204	0.0406	0.0088	0.0783
KRT34	0.0056	0.0029	0.0094	0.0132	0.0312
KRT36	0.0537	0.0437	0.0344	0.0176	0.1494
KRT37	0	0.0029	0.0031	0.0059	0.0119
KRT40	0.0028	0.0029	0	0	0.0057
KRT73	0.0028	0.0029	0.0031	0.0029	0.0118
KRT74	0.0113	0.0175	0.0063	0.0044	0.0394
KRT75	0.0169	0.0058	0.0063	0.0073	0.0364
KRT76	0.0113	0	0	0.0044	0.0157
KRT78	0.596	1.172	5.7688	2.9941	10.5309
KRT79	0.0028	0.0029	0.0031	0.0015	0.0103
KRT80	1.0424	1.8484	4.6063	2.6667	10.1637
KRT81	0.0254	0.0058	0.0094	0.0117	0.0524
KRT85	0	0.0087	0	0.0015	0.0102
KRT86	0.0113	0	0.0031	0.0044	0.0188

KRT222	0.0056	0.0029	0	0	0.0086
KRTAP11-1	0.0113	0.035	0.0938	0.0264	0.1665
KRTAP13-2	0.0169	0.0029	0.0031	0.0162	0.0391
KRTAP1-5	0.0028	0	0	0.0015	0.0043
KRTAP17-1	0.0113	0	0.0094	0	0.0207
KRTAP19-1	0	0	0	0.0029	0.0029
KRTAP2-3	0.0028	0	0	0.0029	0.0058
KRTAP3-1	0.0028	0.0029	0	0	0.0057
KRTAP5-10	0.0169	0.0029	0.0125	0.0044	0.0368
KRTAP5-7	0	0.0029	0	0	0.0029
KRTAP5-8	0.0028	0.0029	0	0	0.0057
KRTAP5-9	0.0169	0.0087	0.0031	0.0088	0.0376
