# Supplementary material for: Molecular stratification of the human fetal vaginal epithelium by spatial transcriptome analysis: Spatial transcriptome analysis of the human fetal vaginal epithelium
Source: Acta Biochim Biophys Sin (Shanghai). 2024 Apr 25;56(10):1521–36. doi: 10.3724/abbs.2024063 (PMC11612642; doi:10.3724/abbs.2024063)
Supplement: 24084supplementary_Figures [file 24084supplementary_Figures.pdf]

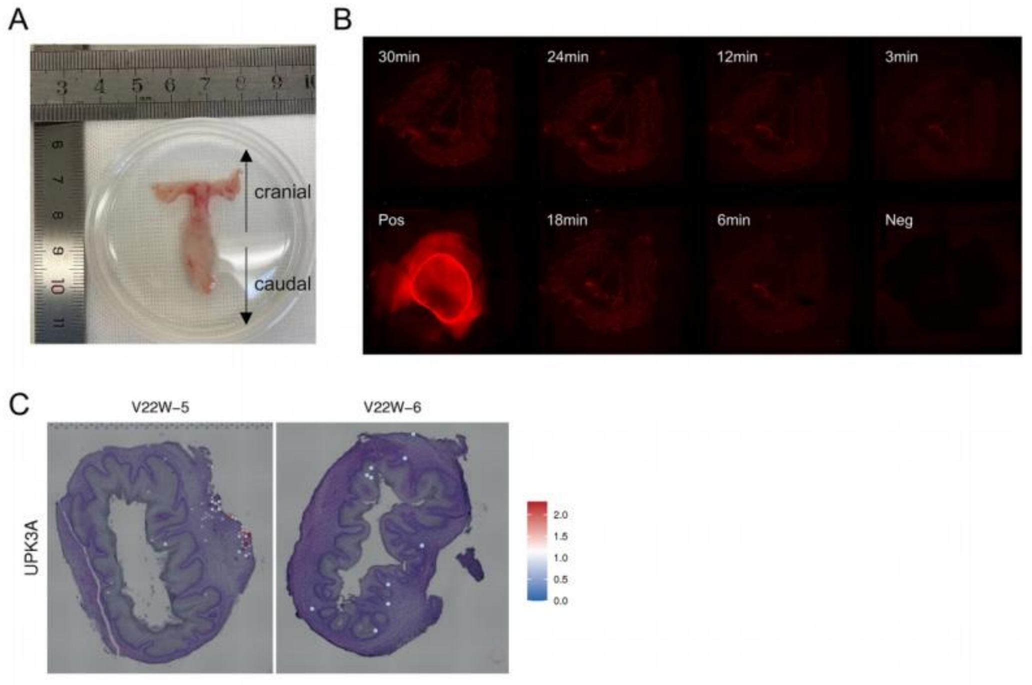

Supplementary Figure S1. Spatial transcriptomics tissue optimization (A) Whole-mount photographs of a human female reproductive tract at 22^+ weeks. (B) Optimization of permeabilization time, with positive (Pos) and negative (Neg) controls. (C) Spatial mapping of UPK3A indicating the region corresponding to the urethra, facilitating the removal of spots around this region to minimize interference from urethral genes.

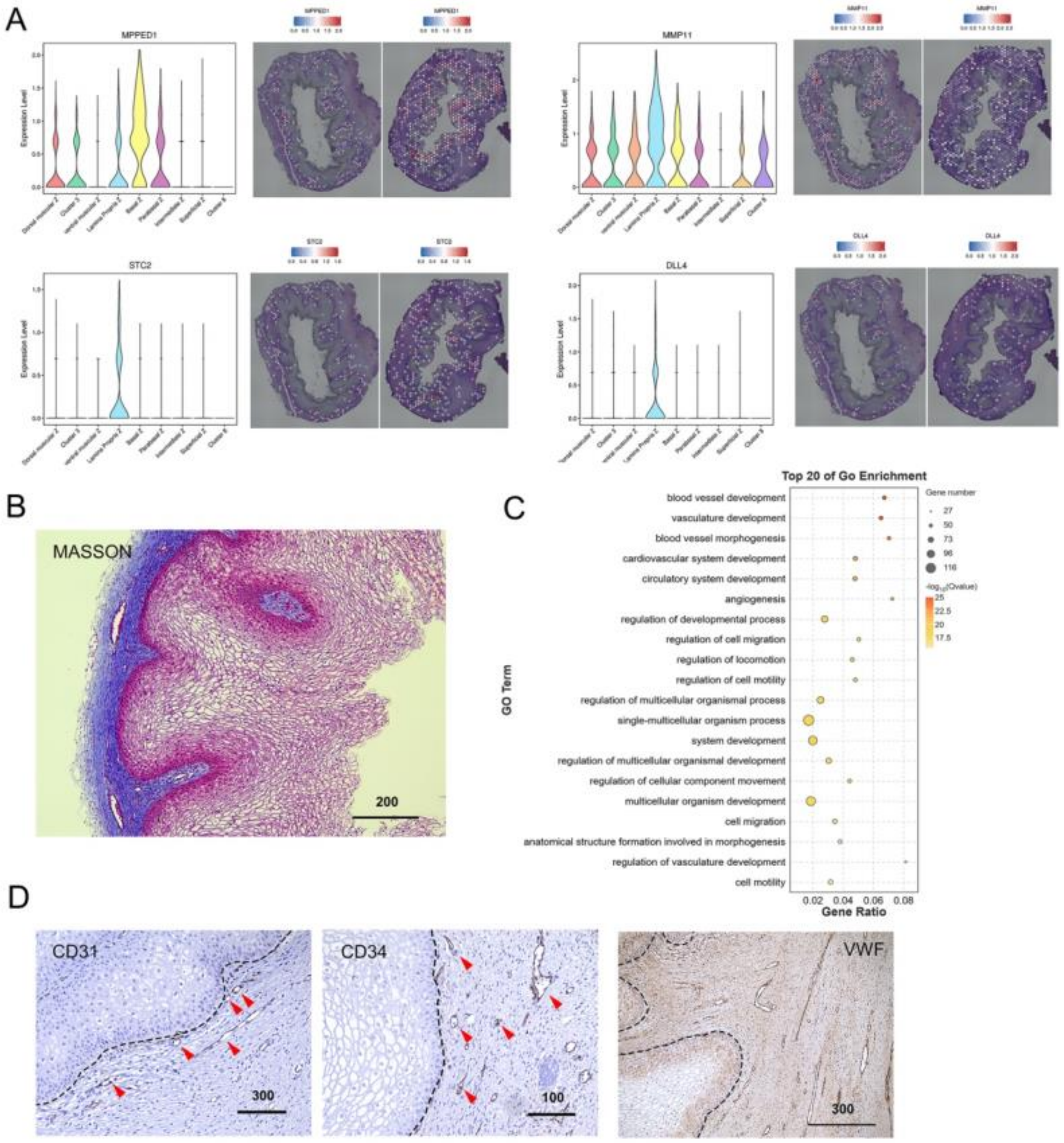

Supplementary Figure S2. Cluster-specific genes expressed in the lamina propria

(A) Violin plot (left) accompanied by spatial mapping (right) of DEGs in the lamina propria, showcasing the spatial distribution of specific molecules. (B) Masson staining of the fetal vaginal wall provides visualization of tissue composition. (C) Bubble plot displaying the top 20 Gene Ontology (GO) enrichments for the lamina propria. (D) Immunohistochemistry (IHC) staining of blood vessel markers CD31, CD34, and VWF in the human fetal vagina, highlighting the vascular structures in the lamina propria.

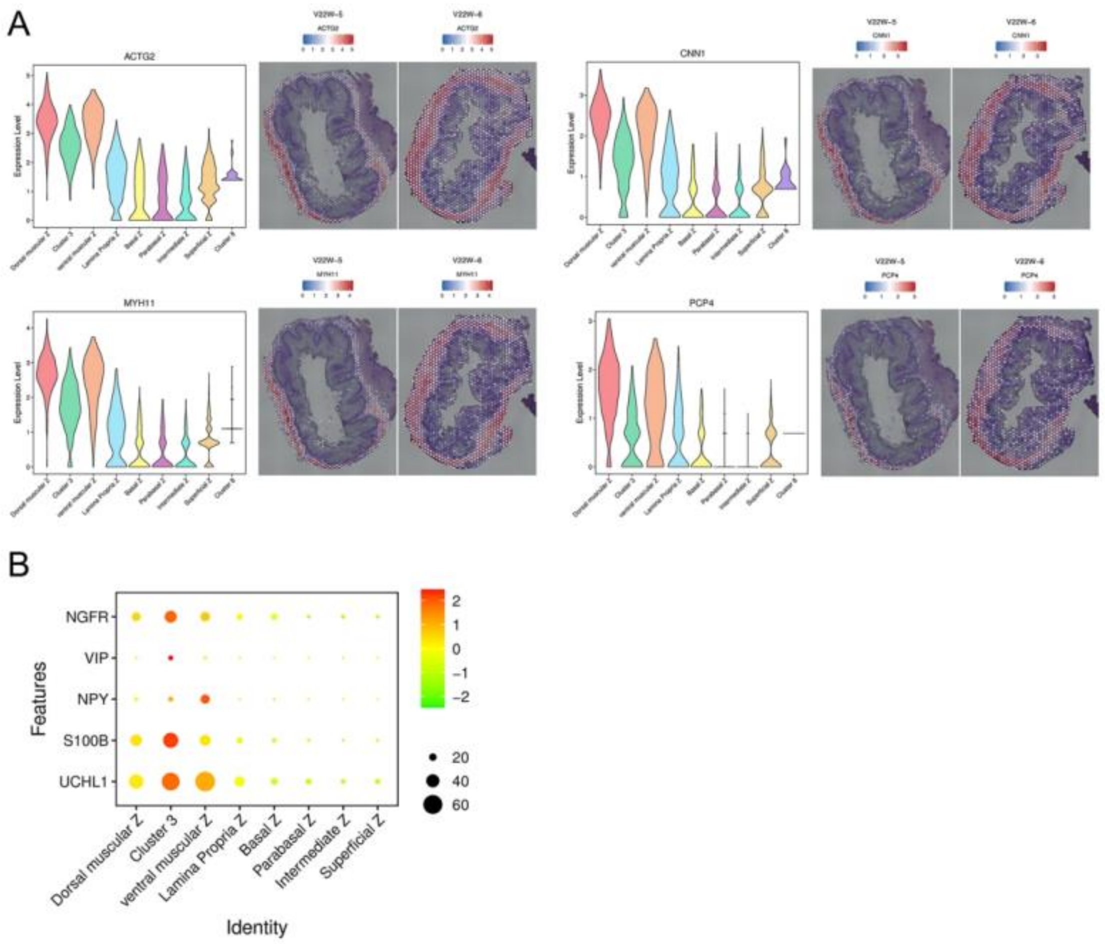

Supplementary Figure S3. Cluster-specific genes expressed in the muscular propria (A) Left: Violin plot depicting differentially expressed genes (DEGs) in the muscular propria. Right: Spatial mapping of these DEGs, illustrating their distinctive spatial distribution within the muscular propria. (B) Plot of neuron-related genes mapped against different vaginal regions, providing insights into their expression patterns in the context of the muscular propria.

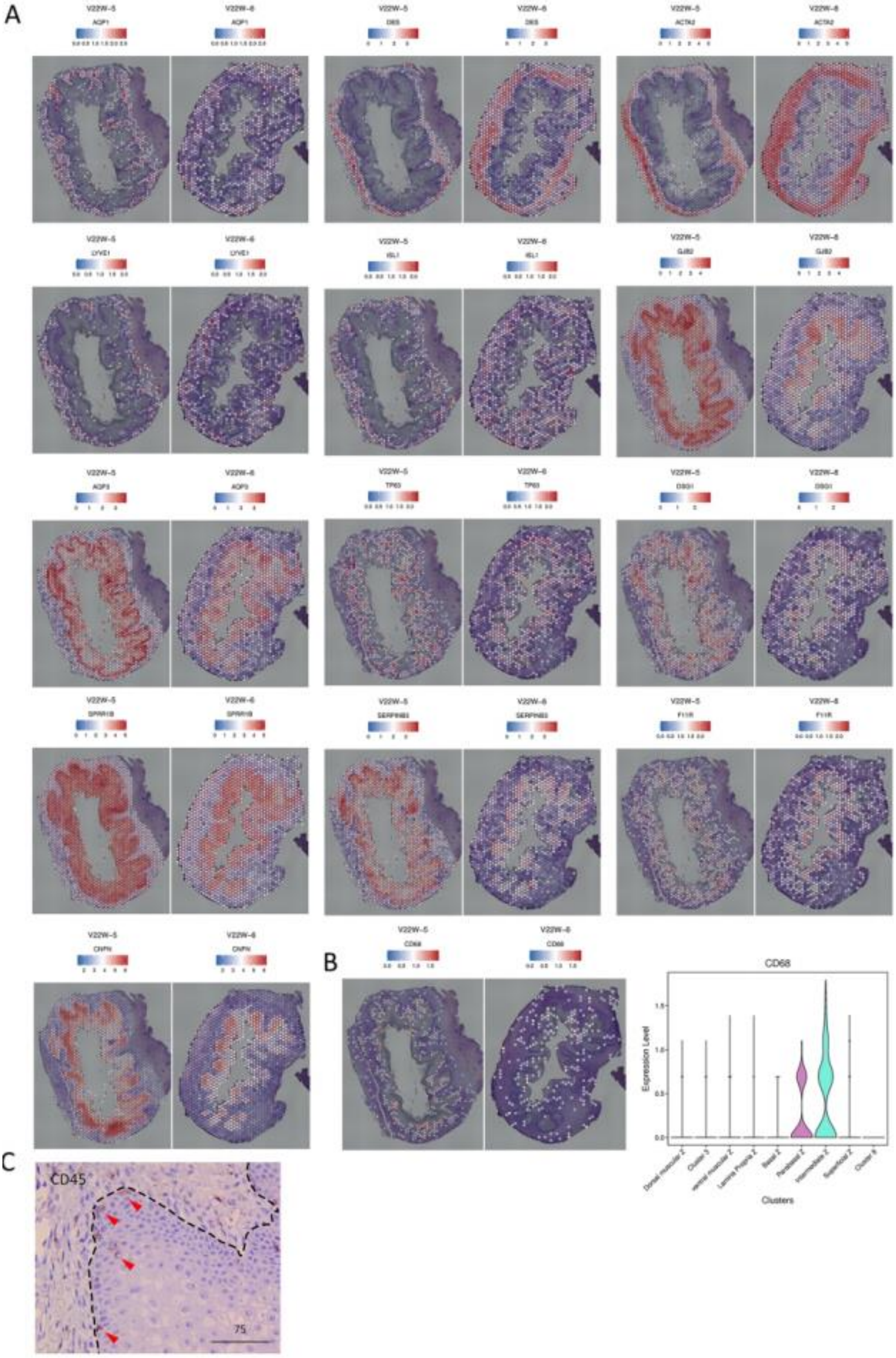

Supplementary Figure S4. Tissue mapping of canonical markers (A) Spatial mapping of canonical markers in the H&E staining slices, providing an overview of

their distribution within the tissue. (B) Dominant expression of CD68 in the parabasal and intermediate zones, as revealed by spatial mapping. (C) Immunohistochemical (IHC) staining depicting the presence of $CD45^+$ cells in the basal zone of epithelia.

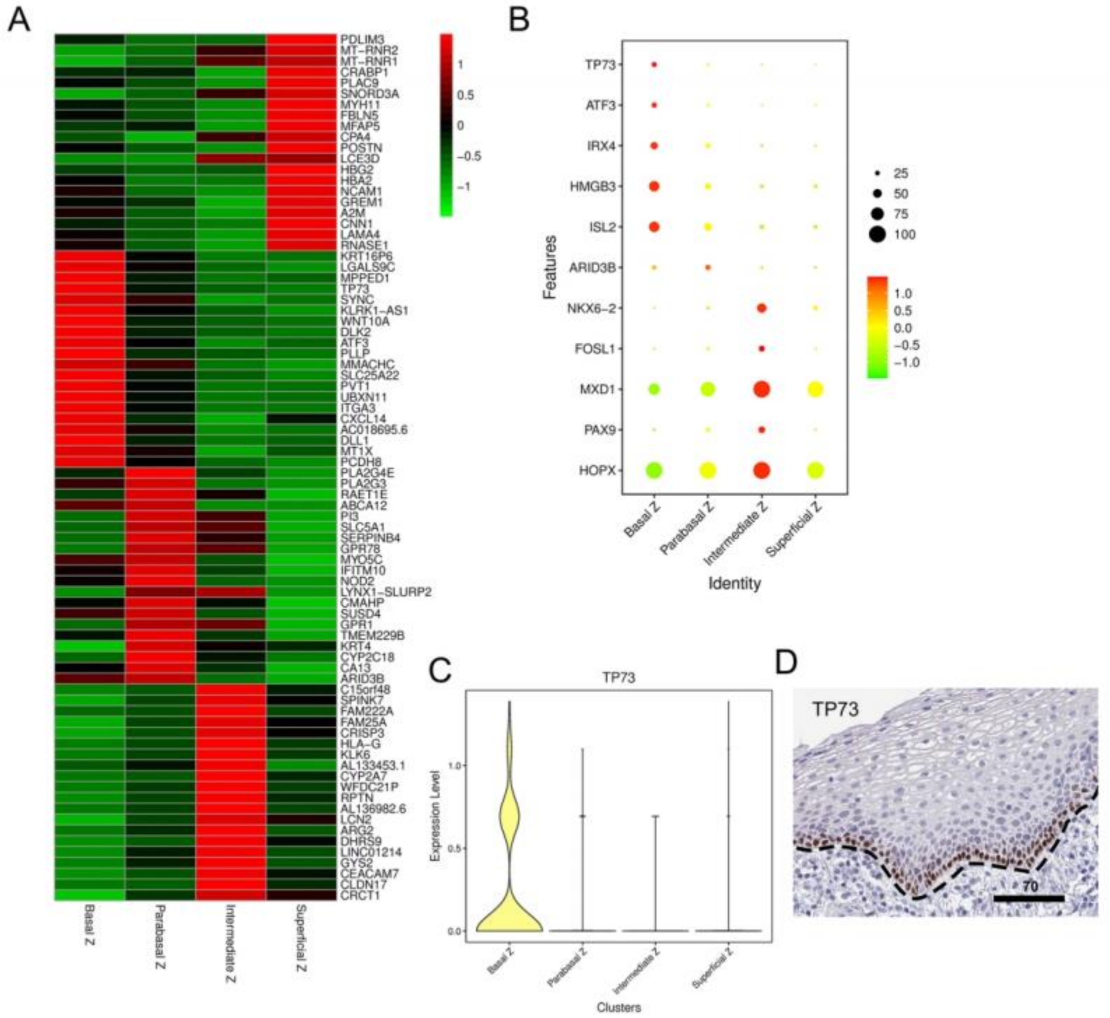

Supplementary Figure S5. Identification of differentially expressed genes in vaginal epithelium (A) Heatmap illustrating the differentially expressed genes (DEGs) among different zones of the vaginal epithelium. For clusters with fewer than 10 genes, all DEGs are displayed. (B) Bubble plot illustrating the top 5 transcription factors (TFs) associated with DEGs in different zones of the vaginal epithelium. All TFs are displayed for clusters with fewer than 5 TFs. (C) Violin plots displaying zone-specific transcription factor TP73 expression, focusing on the basal zone. (D) Immunohistochemical (IHC) staining of TP73 in adult vagina from the Human Protein Atlas (see Methods for details), revealing exclusive expression in the basal layer of the vaginal epithelium.
